# Schottky Emission Distance and Barrier Height Properties of Bipolar Switching Gd:SiOx RRAM Devices under Different Oxygen Concentration Environments

**DOI:** 10.3390/ma11010043

**Published:** 2017-12-28

**Authors:** Kai-Huang Chen, Tsung-Ming Tsai, Chien-Min Cheng, Shou-Jen Huang, Kuan-Chang Chang, Shu-Ping Liang, Tai-Fa Young

**Affiliations:** 1 Department of Electrical Engineering and Computer Science, Tung Fang Design University, Kaohsiung 829, Taiwan; patrick@mail.tf.edu.tw; 2 Department of Materials and Optoelectronic Science, National Sun Yat-Sen University, Kaohsiung 829, Taiwan; tmtsai@faculty.nsysu.edu.tw (T.-M.T.); d9131802@mail.tf.edu.tw (K.-C.C.); 3 Department of Electronic Engineering, Southern Taiwan University of Science and Technology, Tainan 710, Taiwan; 4 Department of Tourism and Leisure Management, Tung Fang Design University, Kaohsiung 829, Taiwan; 5 Department of Mechanical and Electro-Mechanical Engineering, National Sun Yat-Sen University, Kaohsiung 829, Taiwan; u8613319@yahoo.com.tw (S.-P.L.); chiashu.lin@gmail.com (T.-F.Y.)

**Keywords:** nonvolatile memory, gadolinium, RRAM, resistive switching, silicon oxide

## Abstract

In this study, the hopping conduction distance and bipolar switching properties of the Gd:SiOx thin film by (radio frequency, rf) rf sputtering technology for applications in RRAM devices were calculated and investigated. To discuss and verify the electrical switching mechanism in various different constant compliance currents, the typical current versus applied voltage (*I-V*) characteristics of gadolinium oxide RRAM devices was transferred and fitted. Finally, the transmission electrons’ switching behavior between the TiN bottom electrode and Pt top electrode in the initial metallic filament forming process of the gadolinium oxide thin film RRAM devices for low resistance state (LRS)/high resistance state (HRS) was described and explained in a simulated physical diagram model.

## 1. Introduction

For smart memory cards and portable electrical device applications, many nonvolatile memory devices such as the ferroelectric random access memory (FeRAM), magnetic random access memory (MRAM) and phrase change memory (PCM) are widely discussed [1,2,3,4,5,6,7,8]. Because of the integrated circuit (IC) compatibility processes, high operation speed, long retention time, low operation voltage, non-destructive readout and simple metal-insulator-metal-metal (MIM) structure, the various metals doped into silicon-based oxide thin films are widely discussed for applications in resistive random access memory (RRAM) devices [9,10,11,12,13].

Typical resistive switching memory materials for metal doped into silicon-based oxide, complex metal oxide and functional materials thin films were selected and considered. In the initial metallic filament forming process in RRAM devices, the ohmic, Schottky emission, Poole-Frankel and hopping conduction mechanism were simulated and established for the electrical transmission model for low resistance state (LRS)/high resistance state (HRS). However, the hopping conduction distance, activation energy and barrier height of the important electrical conduction mechanism of bipolar resistive switching RRAM devices were not widely investigated and discussed [14,15].

To further discuss the bipolar switching properties of gadolinium-doped SiO_2_ thin films dominated by the interface of the TiN electrode and Gd:SiO_2_ film, the Pt/Gd:SiO_2_/TiN RRAM device was fabricated by virtue of the inertia of the Pt electrode as the top electrode. Besides, the Schottky emission distance trend and barrier height value properties of the electrical conduction mechanism, analyses of the gadolinium-doped influence on Gd:SiO_2_ thin films’ resistive switching behaviors were discussed and explained.

## 2. Material and Methods

For the RRAM device fabrication process, the Gd:SiO_2_ thin films deposited on the TiN/SiO_2_/Si substrate were prepared by co-sputtering using pure silicon dioxide and gadolinium targets. To remove the defects of the oxide target and obtain stable plasma during deposition time, the pre-sputtering time of as-deposited thin film was maintained for 20 min under argon atmosphere. The Gd:SiO_2_ thin film was about 10 nm in thickness. In addition, the sputtering power was the (radio frequency, rf) rf power of 200 W and DC power of 10 W for gadolinium and silicon dioxide targets, respectively.

**Figure 1:**
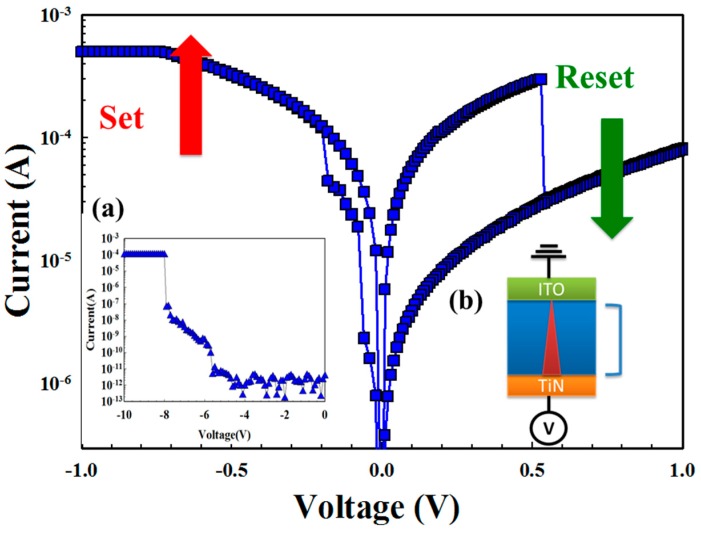
Bipolar switching characteristics of Pt/Gd:SiO_2_/TiN RRAM devices for (**a**) initial forming process and (**b**) MIM structure.

The platinum top electrode for the 200-nm thickness was deposited on gadolinium oxide to form the RRAM devices with the Pt/Gd:SiO_2_/TiN structure. Figure 1b depicts the Gd:SiO_2_ RRAM devices’ structure. The typical current versus applied voltage (*I-V*) characteristics of Gd:SiO_2_ RRAM devices are also measured by the Agilent B1500 semiconductor parameter analyzer (Agilent Technologies, Morris County, NJ, USA). Each experimental parameter was considered and determined by the grey entropy strategy of the situation analysis method. In addition, the *I-V* resistance switching relationship between the Schottky emission distance trend and Schottky barrier height properties of Gd:SiO_2_ thin film RRAM device were obtained and later discussed for different oxygen environments.

## 3. Results and Discussion

The typical *I-V* switching characteristics of the resistance random access memory (RRAM) devices using the gadolinium-doped silicon oxide (Gd:SiO_2_) thin films was observed, and the bipolar behavior was exhibited by applying a base on the TiN electrode and Pt top electrode. To avoid the device burning and being broken due to the high operation current, the compliance current of the RRAM devices was limited to 1 mA. After the initial forming process at a negative applied voltage of 10 V, the RRAM devices reached a low resistance state (LRS) and high resistance state (HRS) in Figure 1. To define the set process, the operation current switching of the RRAM devices was gradual decreased from HRS transferred to LRS by sweeping the negative bias over the set voltage. The RRAM devices from LRS to HRS for applying a large positive bias over the reset voltage were referred to as the reset process. Figure 1 depicts the inverted bipolar switching resistive behaviors of the Gd:SiO_2_ RRAM devices because of its transmission electron in the metallic filament path captured early by the many oxygen vacancies in the ITO top electrode [12].

**Figure 2:**
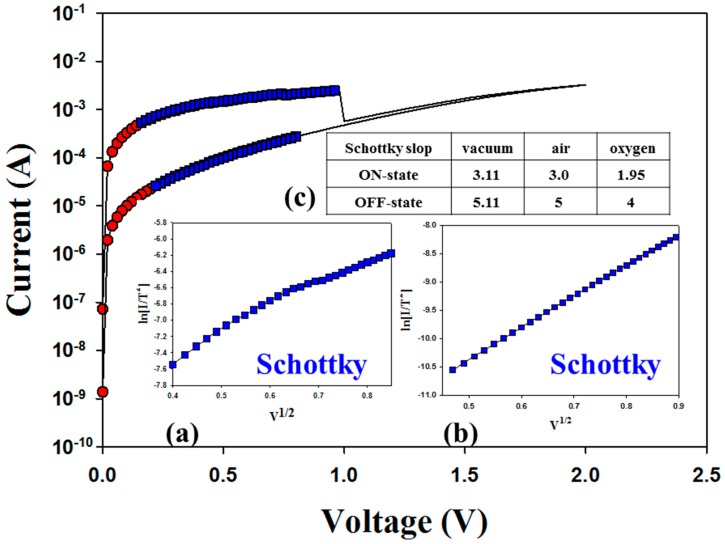
Electrical characteristics of Pt/Gd:SiO_2_/TiN RRAM devices in the plot of ln(I/T^2^) vs. V^1/2^ curves for vacuum environments in: (**a**) low resistance state (LRS); (**b**) high resistance state (HRS); and (**c**) the Schottky slope value for different environments.

**Figure 3:**
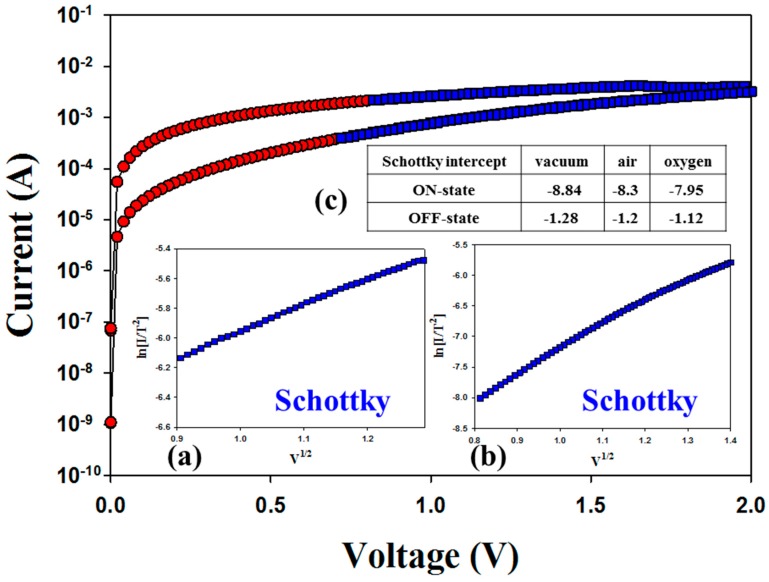
Electrical characteristics of Pt/Gd:SiO_2_/TiN RRAM devices in the plot of ln(I/T^2^) vs. V^1/2^ curves for oxygen environments in: (**a**) LRS; (**b**) HRS; and (**c**) the Schottky slope value for different environments.

Figure 2 and Figure 3 present the *I-V* switching properties of the Gd:SiO_2_ RRAM devices for different vacuum and oxygen environments. For the vacuum environments, the Gd:SiO_2_ thin film RRAM devices for LRS/HRS states all exhibited ohmic conduction in a low electrical field and Schottky emission conduction in a high electrical field. Additionally, the electrical conduction mechanism behavior of Gd:SiO_2_ thin film RRAM devices for LRS/HRS in the oxygen environments was also similar to the vacuum environments. However, different slope and intercept values of the straight line equations of *I-V* switching curves in the vacuum, air and oxygen environments are observed in Figure 2c and Figure 3c. For the on state, the slope value of the Schottky emission conduction in the *I-V* curves of RRAM devices was calculated as 3.11 and 1.95 for the vacuum and oxygen environments, respectively. In addition, the slope value was 5.11 and 4 in the off state for the vacuum and oxygen environments.

For the Schottky conduction mechanism equation,

(1) $$J=A^{*}T^{2}\exp[-q(\Phi_{B}-\sqrt{\frac{qE_{i}}{4\pi \varepsilon_{i}}})/kT]$$

where *T* is the absolute temperature, $\Phi_{B}$ is the Schottky barrier height, *ε_i_* is the insulator permittivity, *K* is Boltzmann’s constant and A* is the Richardson constant. To prove the $\ln\left(\frac{I}{T^{2}}\right)-\sqrt{V}$ relationship curve fitting, the Schottky conduction equation was transferred to:

(2) $$\ln\left(\frac{I}{T^{2}}\right)=\frac{q\sqrt{q/4\pi \varepsilon_{i}d}}{kT}\sqrt{V}-\frac{q\Phi_{B}}{kT}$$

where ($\frac{q\sqrt{q/4\pi \varepsilon_{i}d}}{kT}$) is the slope value and ($\frac{q\Phi_{B}}{kT}$) is the intercept of straight line equations. Therefore, the reciprocal slope value was estimated for the Schottky emission distance trend, and the intercept was around barrier height value for the Schottky conduction equation.

**Figure 4:**
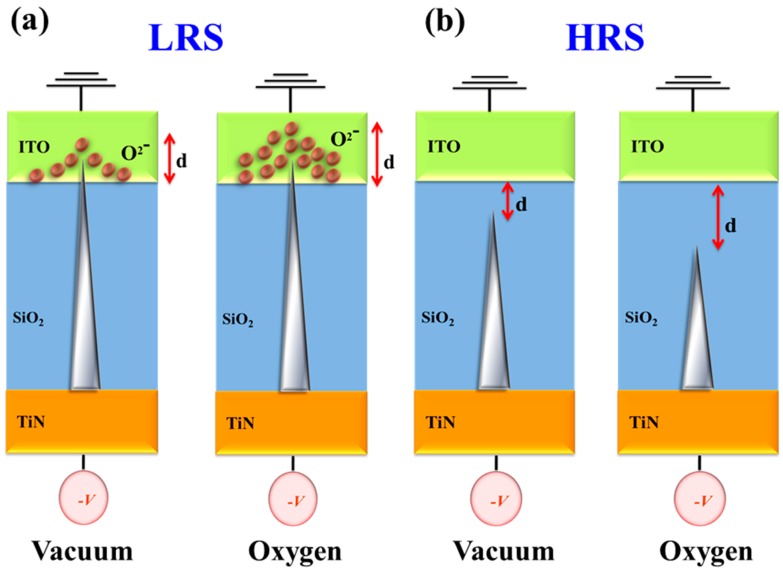
The Schottky emission distance and physical diagram model of the Gd:SiO_2_ RRAM device for (**a**) LRS and (**b**) HRS in different environments.

As presented in Figure 2c and Figure 3c, the Schottky emission distance trend and barrier height value properties of the Schottky emission conduction in the *I-V* curves of RRAM devices for the on/off state were obviously changed for different vacuum, air and oxygen environments. For LRS in vacuum and oxygen environments, the Schottky emission distance model of the RRAM devices is explained and described in Figure 4a. Because of the transmission electrons of the metallic filament path captured early by the many oxygen vacancies in the ITO top electrode for LRS, the Schottky emission distance trend of RRAM devices for the on state was continuously increased for large depletion regions in oxygen environments. In addition, the barrier height value of the Schottky intercept of the RRAM devices in LRS was slightly decreased from 8.84 to 7.95 eV for vacuum and oxygen environments, respectively. In HRS, the Schottky emission distance trend and barrier height value variation were determined by the excess oxygen ions in ITO recombined with the metallic filament paths and transmission to the TiN electrode in vacuum and oxygen environments. In Figure 4b, the short Schottky distance trend of the RRAM devices for the initial positive applied voltage was caused and recombined by less oxygen ions in the metallic filament path oxidation process for vacuum environments. In addition, the Schottky emission distance increases, and the chance for recombination in the metallic filament path by excess oxygen ions in oxygen environments was calculated and observed. Finally, the barrier height value was slightly decreased from 1.28 to 1.18 eV for vacuum and oxygen environments.

## 4. Conclusions

In conclusion, the bipolar switching resistance properties of RRAM devices were fabricated and achieved by doping gadolinium metal into SiO_2_ film in this study. For the different vacuum, air and oxygen environments, the Schottky emission distance trend and barrier height value of the electrical conduction mechanism analyses of Gd:SiO_2_ thin films’ resistive switching behaviors were discussed and explained by the slope and intercept value of straight line equations.

For LRS, the Schottky emission distance trend was continuously increased and caused by large depletion regions of transmission electrons in oxygen environments. In HRS, the Schottky distance trend of the RRAM devices for positive applied voltage was also caused by and recombined in metallic filament oxidation forming for different environments. The similar barrier height value trend of the RRAM devices for different environments was calculated and observed from the Schottky intercept of *I-V* switching curves. The different slope value was inversely proportional to the product of the Schottky distance and material dielectric constant. In addition, the long Schottky emission distance trend was calculated and observed by the small slope value in *I-V* switching curves for high oxygen concentration environments in this study.
